# Haemostasis patterns in patients with acute-on-chronic liver failure and acute decompensation of cirrhosis including thromboelastometric tests with and without the addition of Protac: a pilot study

**DOI:** 10.1186/s12959-022-00438-3

**Published:** 2022-12-13

**Authors:** Andrea Calvo, Miguel Angel Torrente, Klaus Görlinger, Javier Fernandez, Enric Reverter, Julia Vidal, Dolors Tassies, Jordi Colmenero, Annabel Blasi, Juan Carlos Reverter

**Affiliations:** 1 Anaesthesiology and Critical Care Department, Hospital Clínic, Institute d’Investigacions Biomédica AgustPi I Sunyer (IDIBAPS), University of Barcelona, Barcelona, Spain; 2 Haematology Department, Hospital Clínic and University of Barcelona, Barcelona, Spain; 3 Department of Anaesthesiology and Intensive Care Medicine, University Hospital Essen, University Duisburg-Essen, Essen, Germany; 4 Medical Department, Tem Innovations GmbH, Munich, Germany; 5 Institut d’Investigacions Biomèdiques August Pi-Sunyer (IDIBAPS) Y Ciber de Enfermedades Hepáticas Y Digestivas (CIBEREHD), Liver Unit, Institut de Malalties Digestives I Metabòliques, Hospital Clínic and University of Barcelona, Barcelona, Spain; 6 Anaesthesiology Department, Hospital Clínic, Barcelona, Spain; 7 Anaesthesiology Department, Hospital Clínic and University of Barcelona, Spain, Institut d’Investigacions Biomèdiques August Pi-Sunyer (IDIBAPS) Y Ciber de Enfermedades Hepáticas Y Digestivas (CIBEREHD), 08036 Barcelona, Spain

**Keywords:** Cirrhosis, Haemostasis, Thromboelastometry, Thrombomodulin, Protein C, Protac

## Abstract

**Background:**

Thromboelastometry is considered the best method to assesses hemostasis in liver disease. Diagnostic performance could be improved by adding protein C activators such as thrombomodulin or Protac®.

We assessed changes in ROTEM parameters after the addition of Protac® in patients with acute-on-chronic liver failure (ACLF), acute decompensation (AD), and healthy individuals (HI) to define different hemostasis patterns, considering standard and velocity ROTEM parameters, and assess whether Protac® can improve the definition of the pattern.

**Methods:**

Pre-test, we investigated whether diluted EXTEM reagent improved the effect of Protac® on the clotting time (CT)-ratio with and without Protac®. Ten ACLF and 20 AD patients and 21 HI were included in the main study.

**Results:**

Standard EXTEM was used in the main study. INTEM CFT, INTEM A5 (inverse), and INTEM TPI (inverse) were the parameters that best differentiated liver disease from HI (ROC AUC, 0.921, 0.906, and 0.928, respectively; all *P-values* < *0.001*). Combining INTEM CFT with EXTEM LI60-ratio only slightly improved the diagnostic performance (ROC AUC, 0.948; *P* < *0.001*). EXTEM LI60 and INTEM maxV-t were the parameters that best differentiated between ACLF and AD patients (ROC AUC, 0.743, *P* = *0.033*; and 0.723, *P* = *0.050*; respectively). Combining EXTEM LI60 + INTEM maxV-t moderately improved the diagnostic performance (ROC AUC, 0.81, *P* < *0.001*).

**Conclusions:**

ROTEM velocity, fibrinolysis parameters and the indices calculated improve the diagnosis in combination with standard parameters (e.g., CFT and A5). Ratios calculated with and without Protac® (e.g., EXTEM LI60-ratio) only slightly increased the diagnostic performance in discriminating hemostasis patterns.

**Supplementary Information:**

The online version contains supplementary material available at 10.1186/s12959-022-00438-3.

## Introduction

The preservation of the haemostatic capacity in patients with liver disease relies on a balanced reduction in pro- and anticoagulant drivers [1, 2]. To understand the concept of rebalanced haemostasis, knowledge of the methods and limits of the coagulation tests used [3–5]. Standard coagulation tests only mirror the amount of procoagulant factors in plasma, and cannot detect the contribution of cells in the whole blood. The anticoagulant pathway (protein C and S) is not properly examined in any coagulation assay currently used in clinical practice, but rotational thromboelastometry (ROTEM) is considered the best method to assesses haemostasis patterns in patients with liver disease [6–12].

Protein C levels are decreased in patients with cirrhosis [13]. Therefore, the global test, by definition, underestimates the coagulation capacity of blood samples from cirrhotic patients. The protein C system requires the endothelial cell transmembrane protein thrombomodulin (TM) to become activated [14]. The lack of endothelial cells or any protein C activators results in a lack of protein C activation during clot formation [15]. This concept has been extensively investigated using thrombin generation tests, which have been carried out in the presence and absence of a soluble form of TM to allow for protein C activation [16]. Due to low protein C levels, TM-modified thrombin generation does not result in decreased thrombin generation in patients with cirrhosis (TM-resistance), whereas adding thrombomodulin to blood samples from healthy individuals results in significant decreases in thrombin generation [17, 18]. Similar results can be achieved by adding Protac® (Pentapharm, Basel, Switzerland), a snake venom which activates protein C [19–23]. Accordingly, Protac® results in a significant decrease in the endogenous thrombin potential (ETP) of healthy individuals but not patients with cirrhosis. Similarly, in the presence of Protac®, ETP was significantly higher in patients with acute liver failure than in healthy controls [2, 24, 16, 22].

ROTEM assesses global coagulation and clot dynamics in real time through the natural phases of clotting initiation, clot kinetics, and stability until clot breakdown and is performed on whole blood. ROTEM is considered the mainstay of coagulation monitoring during liver transplantation. However, similar limitations may apply for ROTEM regarding the protein C system, which is profoundly altered in patients with liver disease [25]. Thromboelastometry with the addition of thrombomodulin or Protac® has not yet been reported. 

Therefore, the primary end point of this study was to assess changes in ROTEM parameters after the addition of Protac® in patients with acute-on-chronic liver failure (ACLF) and acute decompensation (AD) of cirrhosis and compare these results with healthy individuals.

A secondary end point was to define a different haemostasis pattern, considering both standard and velocity ROTEM parameters in the same groups and to assess whether assays including Protac® improve this pattern definition.

## Material and methods

### Study design

This was a single centre prospective observational study, approved by the Hospital Clinic of Barcelona Research Ethics Committee (approval number HCB/2017/0948) in April 2018. Healthy individuals and patients were enrolled after giving written informed consent. All study procedures were performed following the ethical standards of the Declaration of Helsinki. The study followed the Strengthening the Reporting of Observational Studies in Epidemiology (STROBE) statement guidelines for observational cohort studies [26].

### Study population and data collection

Adult patients admitted to the Hospital Clinic of Barcelona, Spain who were diagnosed with ACLF or AD according to the diagnostic criteria of the Consortium definition [27, 28] and healthy individuals were enrolled between December 1^st^, 2020, and June 18^th^, 2021. Patients with previous coagulation disorders or thrombotic events, those with transfusions of blood products (plasma, platelets, or fibrinogen) within the 7 days before admission, and those taking anticoagulants or platelet aggregation inhibitors were excluded.

### Outcome measurements

Demographic data, the severity of liver disease evaluated using the Child–Pugh score, the Model for End-Stage Liver Disease (MELD), Sequential Organ Failure Assessment (SOFA), and chronic liver failure (CLIF)–SOFA scores were collected. Data on haemorrhagic and thrombotic events and transfusion requirements were collected throughout hospitalization. Bleeding events were defined according to the following criteria: fatal bleeding, symptomatic bleeding in a critical area or organ, and/or bleeding causing a fall in haemoglobin levels of ≥ 2 g/dL or leading to transfusion ≥ 2 units of whole blood or packed red cells [29].

The standard coagulation test (international normalized ratio [INR], aPTT, platelet count, and fibrinogen) and routine laboratory tests also were collected.

### Pre-test study

Initially we analysed blood samples from 10 patients with ACLF, 10 patients with AD, and 10 healthy individuals to assess whether diluted EXTEM reagent (tissue factor activation) improved the effect of Protac® on the clotting time (CT)-ratio with and without Protac®. The pre-test study was based on the hypothesis that ROTEM tests with reduced tissue factor concentration might be more sensitive to decreased thrombin generation, as expected in advanced stages of liver disease [20].

### Sample preparation

ROTEM was performed using EXTEM reagent diluted to different levels by mixing with HEPES buffer: 1:1 (undiluted), 1:10, 1:100, and 1:500 diluted. Protac® vials (3 U) were reconstituted with 200 µl saline (15 U/ml corresponding to 0.3 U /20 µl), and then 20 µl of reconstituted Protac® were added to 300 ml of citrated blood and incubated for 5 min at 37 ºC (on the ROTEM preheating platform) before tissue factor activated tests were started with different EXTEM reagent dilutions.

The CT-ratio with and without Protac® found with the 1:1 undiluted EXTEM reagent was 1.22 ± 0.35 for healthy individuals and 0.99 ± 0.23 for cirrhotic patients. With the dilution of the EXTEM reagent 1:10, the CT-ratio increased from 1.38 ± 0.29 for healthy individuals to 1.17 ± 0.15 for cirrhotic patients. Accordingly, no advantage in performing the test with diluted EXTEM reagent was found. The results did not improve with dilutions of 1:100 and 1:500. Therefore, the main study was carried out with undiluted EXTEM reagent according to the manufacturer’s instructions.

### Main study

Twenty consecutive AD patients, 10 ACLF patients, and 21 healthy individuals were included in the main study. Standard, velocity, fibrinolysis and calculated thromboelastometric indices were used to discriminate between patients with liver disease and healthy individuals and between ACLF and AD patients.

### Thromboelastometry (ROTEM) assays and parameter

ROTEM was performed within one hour after blood sampling. Blood samples were collected in a BD Vacutainer (9NC 0.129 M) through a peripheral venous catheter during the first 24 h of admission. ROTEM was performed with a point-of-care rotational thromboelastometry (ROTEM) *delta* device (TEM Innovations, Munich Germany). Measurements were carried out by extrinsic activation (tissue factor at different concentrations; EXTEM and diluted EXTEM), extrinsic activation with platelet inhibition (tissue factor plus cytochalasin D; FIBTEM), intrinsic activation (ellagic acid without and with heparinase; INTEM and HEPTEM) and running tests with and without Protac® (Pentapharm, Basel, Switzerland), a snake venom extract that activates protein C (PC) [30].

*Standard ROTEM parameters:* clotting time (CT in s), reflecting the initiation of the clotting process; clot formation time (CFT in s), reflecting the propagation of clot formation; time-to-20 mm clot firmness amplitude (TT20 = CT + CFT in s) [31]; alpha angle (ALPHA in °), reflecting clot kinetics; clot firmness amplitude 5 min after CT (A5 in mm), clot firmness amplitude 10 min after CT (A10), and maximum clot firmness (MCF in mm).

*Calculated thrombosis indices:* shear elastic modulus strength (G = 5000 × MCF / (100 – MCF)) and the thrombodynamic potential index (TPI = [(100 × MCF) / (100 – MCF)] / CFT in s^−1^) [32].

*Velocity parameters for the first derivate curve:* area under the first derivative curve until MCF (AUC in mm × 100), maximum velocity (maxV in mm/min), and time to maximum velocity (maxV-T in s).

*Fibrinolysis parameters:* lysis index 30, 45, and 60 min after CT (LI30, LI45 and LI60, reflecting residual clot firmness amplitude in % of MCF at 30, 45, and 60 min after CT), and maximum lysis (ML reflecting the decrease in clot firmness amplitude in % of MCF during run time).

The first derivative of the thromboelastometric curve (velocity curve) described by Sorensen et al*.* shows a profile similar to the thrombin generation curve [33]. Here, the ROTEM velocity parameter maxV-t corresponds to the thrombin burst rate (time to peak (ttP)), maxV to the thrombin generation peak (TPi), and AUC to the total amount of thrombin generated (endogenous thrombin potential (ETP)). CalcuRo software (Tem Innovations, Munich, Germany) was used to evaluate the rate of tensile strength exerted during clot formation.

### Definition of hypocoagulable profile

The presence of ≥ 3 parameters outside the reference range in patients with both EXTEM and INTEM tests (prolonged CT or CFT, decreased alpha angle, and decreased MCF) and ≥ 2 parameters in those with only the EXTEM test [19].

### Definition of a hypercoagulable profile

The presence of ≥ 2 of the following: shortened CT or CFT, increased alpha angle, or increased MCF [19].

### Statistical analysis

No sample size was calculated for this pilot study. Continuous variables were expressed as median (interquartile range), and categorical variables as numbers (percentages). Missing data was minimal (below 0.5%) and missing observations were not imputed. Comparisons between groups were conducted with either the Fisher Exact test or the Mann–Whitney test. Receiver operating characteristic (ROC) curve analyses were made to differentiate patients with liver disease from healthy subjects, using ROTEM parameters which were significantly different between the two groups.

Statistical significance was established as 0.05. All analyses were performed using the Statistical Package for Social Sciences, version 22.0(SPSS, Chicago, IL) and the level of significance was established as 5% in a two-sided test.

## Results

Demographic data of healthy controls and patients with liver disease are presented in Table 1. Alcohol was the most frequent aetiology in all patients. Sixty percent of patients with acute-on-chronic liver failure (ACLF) were grade 3, mainly due to haemodynamic, renal and liver failure. Four patients with acute decompensation of cirrhosis (AD) and 3 with ACLF had a bleeding event related to portal hypertension during hospitalization. No patient had thrombotic events. One patient with ACLF died after 2 weeks of admission due to sepsis complicated by multiorgan failure.

**Table 1:** Demographic data of liver patients and healthy controls (medians and interquartile ranges)

**Demographic data**	**Healthy Controls (CON; *N* = 21)**	**Acute Decompensation (AD; *N* = 20)**	**Acute-On-Chronic Liver Failure (ACLF; *N* = 10)**
Age [years]	53 (48–70)	62 (51–68)	56 (52–67)
Sex [male/female]	10/12	14/6	7/3
Child [A/B/C]	-	1/15/4	1/1/8
Aetiology of liver disease [OH/HCV/NASH/others]	-	8/4/6/2	7/1/2/0
MELD Score	-	18 (14–24)	26 (19–30)
Haemoglobin [g/dL]	13.1 (12.3–14.0)	9.6 (8.8–9.7)	9.1 (7.3–10.0)
ACLF grade^a^ [1/2/3]	-	-	2/2/6
Creatinine [mg/dL]	-	1.1 (0.7–2.7)	2.2 (1.0–4.2)
Na [mmol/L]	-	136 (134–139)	137 (134–140)
K [mmol/L]	-	3.8 (3.5–4.1)	4 (3.6–4.4)
Albumin [g/L]	-	32 (23–33)	24 (23–29)
Bilirubin [mg/dL]	-	2.3 (1.5–3.4)	9 (2–22)
PT [Quick; %]	97(96–99)	49 (46–58)	37 (33–46)
APTT [sec]	25(23–29)	32 (29–35)	39 (31–50)
Platelet count [nL^−1^]	210(158–250)	65 (55–101)	46 (33–98)
Fibrinogen [g/L]	3.3(3–3.9)	2.3 (1.9–2.6)	1.9 (1.4–2.9)

^a^At the time of blood sampling

### Pre-test study: Diluted EXTEM reagent did not improve the diagnostic performance of the effect of Protac® on clotting time (CT)-ratio (with and without Protac®)

Accordingly, the main study was carried out with the undiluted EXTEM reagent according to the manufacturer’s instructions (see methods section).

### Samples from patients with cirrhosis showed a hypocoagulable profile in standard thromboelastometry compared with healthy individuals

Thromboelastometry performed in standard conditions showed a hypocoagulable profile in 55% of patients with chronic liver disease (CLD) compared with O% in healthy individuals. Kinetic parameters, such as CT and CFT, were significantly prolonged in patients with CLD. Maximum clot firmness (MCF) was significantly lower in CLD patients compared with controls. Overall, there were no significant differences in thromboelastometry parameters across the severity of liver disease. LI60 in EXTEM was significantly higher in patients with ACLF vs. controls: 99 (96–100) vs. 93 (90–96), *P* < *0.01.* Velocity parameters from the first derivative curve were in agreement with standard thromboelastometric parameters, with a lower AUC and maximum velocity (maxV) in CLD patients compared with controls, whereas the time to reach maximum velocity (maxV-t) showed more variability (Table 2).

**Table 2:** Standard thromboelastometric tests in patients with chronic liver disease and healthy controls (anova with bonferroni adjustment)

	**Healthy Controls (CON; a; *N* = 21**	**Acute Decompensation (AD; b; *N* = 20**	**Acute-On-Chronic Liver Disease** **(ACLF; c; *N* = 10**	***P*-values**
**EXTEM** **(Reference Ranges)**				
CT (38–79 s)	55 (51–60)	62 (56–74)	71 (64–77)	< 0.05, a vs. b and c
CFT (34–159 s)	80 (72–92)	146 (100–181)	139 (94–214)	< 0.05, a vs. b and c
TT20 (CT + CFT)	141 (126–149)	221 (159–255)	205 (168–281)	< 0.01, a vs. b and c
ALPHA (63–83˚)	74 (71–75)	67 (59–74)	65 (56–71)	< 0.05, a vs. b and c
A5	48 (43–52)	32 (28–38)	30 (19–39)	< 0.01, a vs. b and c
A10 (43–65 mm)	58 (54–60)	43 (37–48)	44 (35–54)	< 0.01, a vs. b and c
MCF (50–72 mm)	65 (63–67)	52 (48–57)	53 (46–57)	< 0.01, a vs. b and c
G	9108 (8534–10,267)	5402 (4661–6759)	5923 (4476–7880)	< 0.01, a vs. b and c
TPI	2.33 (1.82–2.81)	0.69 (0.53–1.30)	0.84 (0.42–1.37)	< 0.01, a vs. b and c
**INTEM** **(Reference Ranges)**				
CT (100–240 s)	182 (167–184)	190 (163–218)	215 (194–247)	ns
CFT (30–110 s)	67 (61–83)	138 (103–172)	136 (93–213)	< 0.01, a vs. b and c
TT20 (CT + CFT)	255 (239–275)	326 (306–349)	369 (312–442)	< 0.01, a vs. b and c
ALPHA (70–83˚)	76 (73–77)	67 (60–71)	62 (55–70)	< 0.01, a vs. b and c
A5	46 (42–49)	33 (28–37)	32 (24–38)	< 0.01, a vs. b and c
A10 (44–66 mm)	57 (54–59)	43 (37–47)	41 (33–47)	< 0.01, a vs. b and c
MCF (50–72 mm)	62 (61–65)	50 (45–56)	51 (43–55)	< 0.01, a vs. b and c
G	8322 (7988–9442)	5278 (4313–6558)	5192 (3918–6360)	ns
TPI	2.43 (1.79–3.04)	0.72 (0.51–1.11)	0.80 (0.35–1.30)	< 0.01, a vs. b and c
CT-Ratio (INTEM/HEPTEM)	1.04 (0.95–1.08)	0.96 (0.89–1.07)	1.03(1.01–1.25)	ns
**FIBTEM** **(Reference Ranges)**				
CT	52 (49–57)	61 (50–67)	61 (51–77)	ns
A5	16 (12–17)	14 (10–16)	10 (8–14)	ns
A10 (7–23 mm)	16 (13–19)	14 (11–16)	10 (8–16)	ns
MCF (9–25 mm)	17 (14–19)	15 (12–18)	11 (9–17)	ns
**Velocity parameter**				
EXTEM AUC	6446 (6263–6731)	5152 (4777–5692)	5405 (4705–6049)	< 0.01, a vs. b and c
EXTEM maxV	15 (13–17)	11 (8–14)	10 (8–14)	< 0.01, a vs. b and c
EXTEM maxV-t	103 (73–118)	92 (63–145)	105 (85–129)	ns
FIBTEM AUC	1699 (1409–2161)	1532 (1206–1817)	1095 (965–1601)	ns
FIBTEM maxV	14 (11–16)	10 (8–16)	8 (6–11)	ns
FIBTEM maxV-t	58 (53–69)	66 (61–77)	72 (60–80)	ns
INTEM AUC	6257 (6082–6427)	5178 (4662–5687)	5241 (4395–5618)	< 0.01, a vs. b and c
INTEM maxV	18 (15–20)	11 (9–13)	10 (7–13)	< 0.01, a vs. b and c
INTEM maxV-t	205 (193–224)	205 (180–243)	254 (214–288)	0.05, b vs. c
**Fibrinoliysis parameter**				
EXTEM LI30 (94–100%)	100 (100–100)	100 (100–100)	100 (100–100)	ns
EXTEM LI45	97 (96–98)	99 (96–100)	100 (99–100)	< 0.05 a vs. c
EXTEMLI60 (86–100%)	93 (90–96)	96 (91–98)	99 (96–100)	< 0.05 a vs. c
EXTEM ML (< 15% at 60 min after CT)	7.0 (4.0–9.0)	4.0 (2.0–8.5)	1.0 (0.0–3.2)	< 0.05 a vs. c
FIBTEM LI30	100 (100–100)	100 (99–100)	100 (97–100)	ns
FIBTEM LI45	100 (100–100)	100 (98–100)	100 (97–100)	ns
FIBTEM LI60	100 (99–100)	100 (97–100)	100 (96–100)	ns
FIBTEM ML	0 (0–0.5)	0 (0–2.5)	0 (0–3.5)	ns
ΔLI60 (FIBTEM – EXTEM)	6(4–9)	2.5(1–6.7)	0.5(-2.7–3.2)	< 0.05 a vs. c

### Healthy individuals, but not patients with cirrhosis, showed a pattern of hypocoagulability in thromboelastometry performed after the addition of Protac® to the blood sample

ROTEM kinetic parameters were prolonged when Protac® was added to blood samples from healthy subjects: TT20 EXTEM, 141 (126–149) vs. 163 (140–183) s*, P* = *0.003* and INTEM CFT, 67 (61–83) vs. 81 (72–97) s, *P* = *0.009*. This effect was less pronounced in patients with AD cirrhosis (only INTEM CT increased from (190 (163–218) to 212 (166–237) s, *P* = *0.02*) after adding Protac®, whereas in patients with ACLF the opposite was observed: CFT, 136 (93–213) vs. 119 (76–207) s, *P* = *0.02*, and TPI, 0.80 (0.35–1.30) vs. 1.01 (0.34–1.50) s^−1^, *P* = *0.01*, without and with Protac®, respectively (Table 3). Few variations were seen in velocity parameters in healthy controls and in patients after the Protac® challenge. EXTEM LI60 significantly increased with Protac® compared with without Protac® in healthy subjects and in patients with AD. No changes were observed in FIBTEM LI60 run in parallel (Table 3).

**Table 3:** Thromboelastometric tests without and with Protac® challenge in patients with chronic liver disease and healthy controls

	**Without Protac**	**With Protac**	***P*-value**	**Ratio w/who Protac**
**Healthy controls (*N* = 21)**				
**EXTEM**				
CT	55 (51–60)	66 (53–77)	0.02	1.2
CFT	80 (72–92)	92 (80–108)	0.01	1.1
TT20 (CT + CFT)	141 (126–149)	163 (140–183)	0.003	1.1
ALPHA	74 (71–75)	72 (69–74)	0.03	0.9
A5	48 (43–52)	44 (41–48)	0.01	0.9
A10	58 (54–60)	55 (53–59)	0.02	0.9
MCF	65 (63–67)	65 (62–68)	0.59	1.0
G	9108 (8534–10,267)	9511 (8507–10,735)	0.25	1.0
TPI	2.33 (1.82–2.81)	2.04 (1.54–2.61)	0.17	0.9
**INTEM**				
CT	182 (167–194)	180 (157–199)	0.66	0.9
CFT	67 (61–83)	81 (72–97)	0.009	1.1
CT + CFT (TT20)	255 (239–275)	255 (247–288)	0.38	1.0
ALPHA	76 (73–77)	73 (71–75)	0.03	0.9
A5	46 (42–49)	45 (43–48)	0.36	0.9
A10	57 (54–59)	56 (53–58)	0.43	0.9
MCF	62 (61–65)	62 (60–66)	0.57	1.0
G	8322 (7988–9442)	9210 (8193–9797)	0.28	1.0
TPI	2.43 (1.79–3.04)	2.02 (1.68–2.34)	0.10	0.9
**FIBTEM**				
CT	52 (49–57)	59 (54–67)	0.01	1.1
A5	16 (12–17)	14 (13–16)	0.22	0.9
A10	16 (13–19)	15 (14–18)	0.42	0.9
MCF	17 (14–19)	16 (15–19)	0.57	0.9
**Velocity parameter**				
EXTEM AUC	6446 (6263–6731)	6518 (6262–6819)	0.54	1.0
EXTEM maxV	15 (13–17)	14 (11–17)	0.25	0.9
EXTEM maxV-t	103 (73–118)	127 (87–146)	0.03	1.2
FIBTEM AUC	1699 (1409–2161)	1601 (1463–1798)	0.09	0.9
FIBTEM maxV	14 (11–16)	12 (9–15)	0.19	0.9
FIBTEM maxV-t	58 (53–69)	74 (59–85)	0.06	1.3
INTEM AUC	6257 (6082–6427)	6454 (6278–6657)	0.02	1.0
INTEM maxV	18 (15–20)	17 (15–18)	0.21	1.3
INTEM maxV-t	205 (193–224)	229 (199–250)	0.23	1.0
**Fibrinolysis parameter**				
EXTEM LI30	100 (100–100)	100 (100–100)	0.10	1.0
EXTEM LI45	97 (96–98)	100 (98–100)	< 0.001	1.0
EXTEM LI60	93 (90–96)	98 (95–99)	< 0.001	1.0
EXTEM ML	7 (4–9)	2 (1–5)	< 0.001	0.3
FIBTEM LI30	100 (100–100)	98 (100–100)	0.12	0.9
FIBTEM LI45	100 (100–100)	97 (100–100)	0.10	0.9
FIBTEM LI60	100 (99–100)	100 (100–100)	0.53	0.9
FIBTEM ML	0 (0–0.5)	0 (0–3.5)	0.53	0.8
**Acute Decompensated Patients (AD; *N* = 20)**				
**EXTEM**				
CT	62 (56–74)	63 (55–75)	0.67	1.0
CFT	146 (100–181)	136 (113–169)	0.49	1.0
TT20 (CT + CFT)	221 (159–255)	198 (173–248)	0.77	1.0
ALPHA	67 (59–74)	67 (63–70)	0.54	1.0
A5	32 (28–38)	33 (29–38)	0.60	1.0
A10	43 (37–48)	44 (39–47)	0.46	0.9
MCF	52 (48–57)	52 (48–56)	0.61	0.9
G	5402 (4661–6759)	5624 (4738–6519)	0.50	1.0
TPI	0.69 (0.53–1.30)	0.80 (0.61–1.04)	0.27	1.0
**INTEM**				
CT	190 (163–218)	212 (166–237)	0.02	1.1
CFT	138 (103–172)	106 (88–152)	0.067	0.9
TT20 (CT + CFT)	326 (306–349)	312 (278–348)	0.73	1.0
ALPHA	67 (60–71)	70 (62–72)	0.43	1.0
A5	33 (28–37)	35 (29–38)	0.79	1.0
A10	43 (37–47)	44 (39–47)	0.74	1.0
MCF	50 (45–56)	50 (47–56)	0.72	1.0
G	5278 (4313–6558)	5595 (4445–6604)	0.61	1.0
TPI	0.72 (0.51–1.11)	1.02 (0.59–1.33)	0.48	1.5
**FIBTEM**				
CT	61 (50–67)	65 (58–72)	0.03	1.0
A5	14 (10–16)	12 (8–16)	0.06	0.8
A10	14 (11–16)	13 (9–17)	0.07	0.9
MCF	15 (12–18)	13 (9–17)	0.11	0.9
**Velocity parameter**				
EXTEM AUC	5152 (4777–5692)	5191 (4851–5590)	0.47	0.9
EXTEM maxV	11 (8–14)	10 (9–12)	0.35	0.9
EXTEM maxV-t	92 (63–145)	85 (68–155)	0.38	1.2
FIBTEM AUC	1532 (1206–1817)	1340 (841–1652)	0.01	0.8
FIBTEM maxV	10 (8–16)	10 (8–14)	0.08	0.8
FIBTEM maxV-t	66 (61–77)	69 (64–79)	0.40	1.0
INTEM AUC	5178 (4662–5687)	5239 (4976–5670)	0.57	0.9
INTEM maxV	11 (9–13)	11 (10–13)	0.51	1.0
INTEM maxV-t	205 (180–243)	215 (196–256)	0.05	1.0
**Fibrinolysis parameter**				
EXTEM LI30	100 (100–100)	100 (100–100)	0.31	1.0
EXTEM LI45	99 (96–100)	100 (99–100)	0.06	1.0
EXTEM LI60	96 (91–98)	97 (95–98)	0.02	1.0
ML EXTEM	4.0 (2.0–8.5)	2.5 (2.0–4.7)	0.02	0.7
FIBTEM LI30	100 (99–100)	100 (100–100)	0.49	0.9
FIBTEM LI45	100 (98–100)	100 (100–100)	0.48	0.9
FIBTEM LI60	100 (97–100)	100 (98–100)	0.48	0.9
ML FIBTEM	0 (0–2.5)	0 (0–1.5)	0.48	0.5
**Acute-On-Chronic Liver Failure Patients (ACLF, *N* = 10)**				
**EXTEM**				
CT	71 (64–77)	64 (54–76)	0.28	0.9
CFT	139 (94–214)	159 (98–251)	0.95	1.0
TT20 (CT + CFT)	205 (168–281)	221 (160–334)	0.50	1.0
ALPHA	65 (56–71)	62 (57–72)	0.85	1.0
A5	30 (19–39)	35 (22–47)	0.41	0.9
A10	44 (35–54)	41 (31–52)	0.72	0.9
MCF	53 (46–57)	51 (41–60)	0.85	1.0
G	5923 (4476–7880)	5380 (3617–7748)	0.50	0.9
TPI	0.84 (0.42–1.37)	0.69 (0.29–1.64)	0.87	1.1
**INTEM**				
CT	215 (194–247)	212 (187–238)	0.61	0.9
CFT	136 (93–213)	119 (76–207)	0.02	0.9
TT20 (CT + CFT)	369 (312–442)	331 (260–458)	0.16	0.9
ALFA	62 (55–70)	67 (61–72)	0.04	1.0
A5	32 (24–38)	33 (24–40)	0.59	1.0
A10	41 (33–47)	41 (32–49)	0.71	0.9
MCF	51 (43–55)	51 (41–56)	0.71	0.9
G	5192 (3918–6360)	5144 (3541–6784)	0.72	1.0
TPI	0.80 (0.35–1.30)	1.01 (0.34–1.50)	0.01	1.1
**FIBTEM**				
CT	61 (51–77)	65 (54–85)	0.30	1.1
A5	10 (8–14)	10 (6–15)	0.36	0.9
A10	10 (8–16)	11 (6–16)	0.57	0.9
MCF	11 (9–17)	12 (9–18)	0.59	1.9
**Velocity parameter**				
EXTEM AUC	5405 (4705–6049)	5145 (4194–6064)	0.57	0.9
EXTEM maxV	10 (8–14)	9 (7–13)	0.85	1.0
EXTEM maxV-t	105 (85–129)	103 (57–144)	0.95	1.1
FIBTEM AUC	1095 (965–1601)	1244 (712–1776)	0.87	1.0
FIBTEM maxV	8 (6–11)	10 (4–15)	1.00	1.0
FIBTEM maxV-t	72 (60–80)	71 (63–89)	0.33	1.1
INTEM AUC	5241 (4395–5618)	5028 (4140–5719)	0.50	0.9
INTEM maxV	10 (7–13)	9 (7–15)	0.30	1.0
INTEM maxV-t	254 (214–288)	265 (222–286)	0.67	0.9
**Fibrinolysis parameter**				
EXTEM LI30	100 (100–100)	100 (100–100)	1.00	1.0
EXTEM LI45	100 (99–100)	100 (99–100)	0.10	1.0
EXTEM LI60	99 (96–100)	99 (97–100)	0.04	1.0
EXTEM ML	1.0 (0.0–3.2)	0.5 (0.0–2.2)	0.02	0.4
FIBTEM LI30	100 (97–100)	100 (94–100)	0.71	0.9
FIBTEM LI45	100 (97–100)	100 (90–100)	0.46	0.9
FIBTEM LI60	100 (96–100)	100 (96–100)	0.71	0.9
FIBTEM ML	0.0 (0.0–3.5)	0.0 (0.0–14)	0.71	0.5

### Differences in standard, velocity, and fibrinolysis thromboelastometric parameters were observed between CLD patients and healthy controls

**Fig.1:**
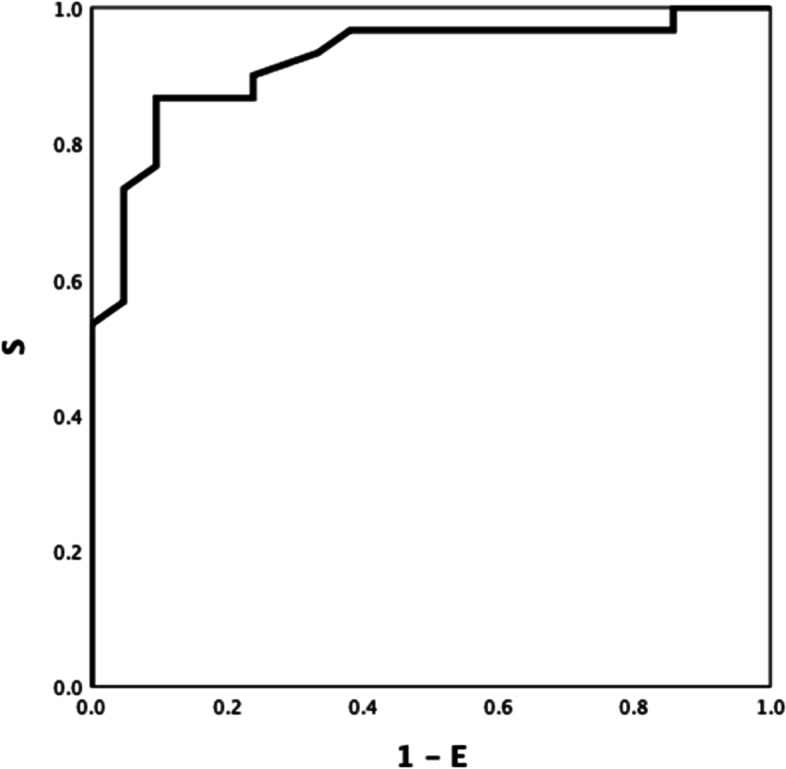
HEALTHY VS DISEASE. INTEM CFT 0.921

**Fig. 2:**
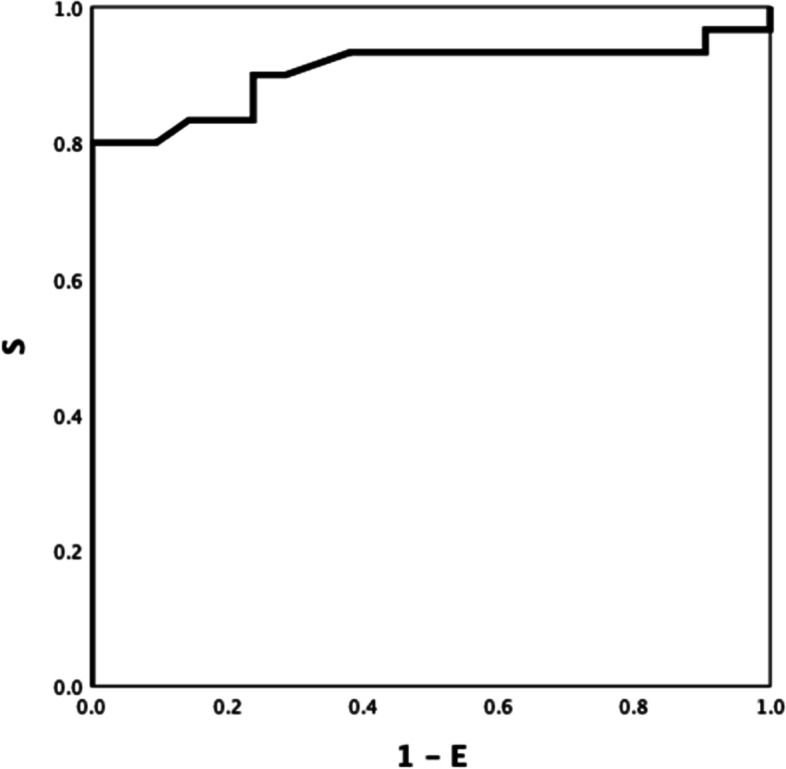
HEALTHY VS DISEASE. INTEM A5 (inverse) 0.906

**Fig. 3:**
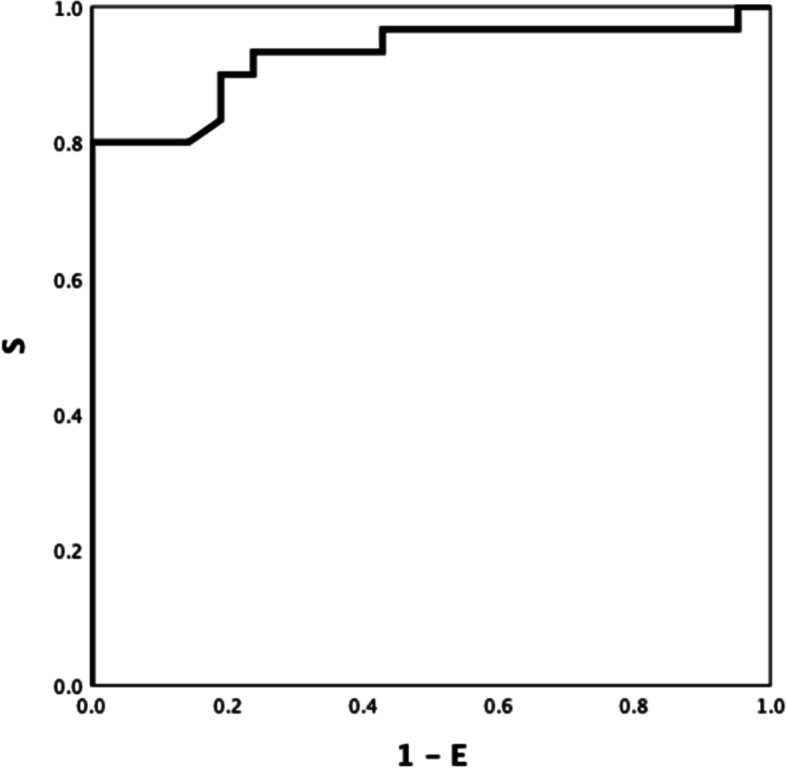
HEALTHY VS DISEASE INTEM TPI (inverse) 0.928

Between-group ROC curve analyses using ROTEM parameters differed significantly different (Table S1 Electronic Supplement), INTEM CFT, INTEM A5 (inverse) and INTEM TPI (inverse) showed the highest ROC AUC: 0.921(Fig. 1), 0.906 (Fig. 2), and 0.928 (Fig. 3) respectively (all *P-values* < *0.001*). INTEM CFT had the highest sensitivity (90%) and INTEM A5 (inverse) the highest specificity (100%). Overall, INTEM TPI (inverse) performed best as it includes CFT and A5 (sensitivity 80%, specificity 100%, PPV 100%, NPV 78%).

**Fig. 4:**
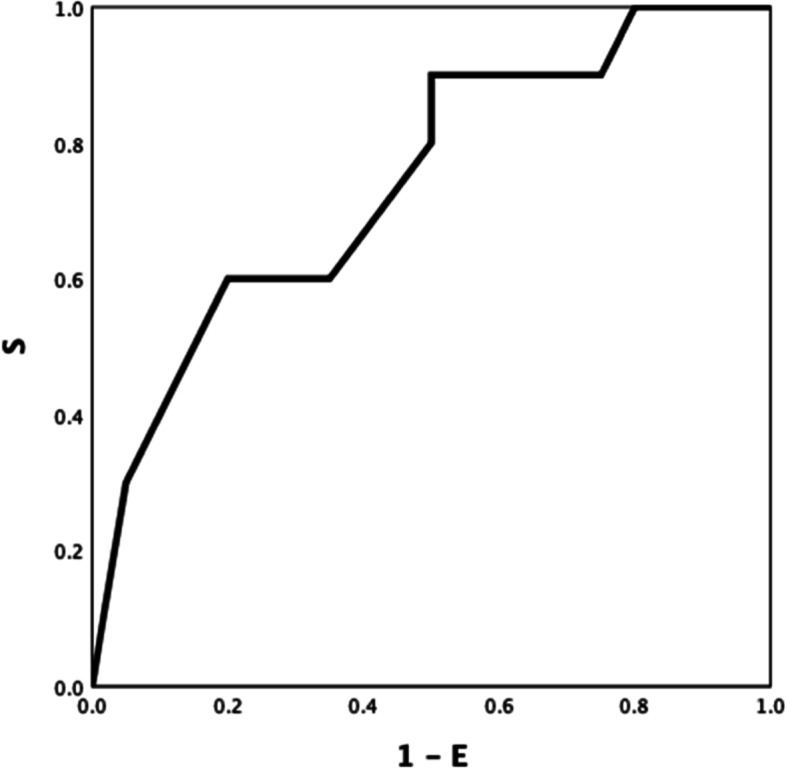
AD VS ACLF EXTEM LI60 0.743

**Fig. 5:**
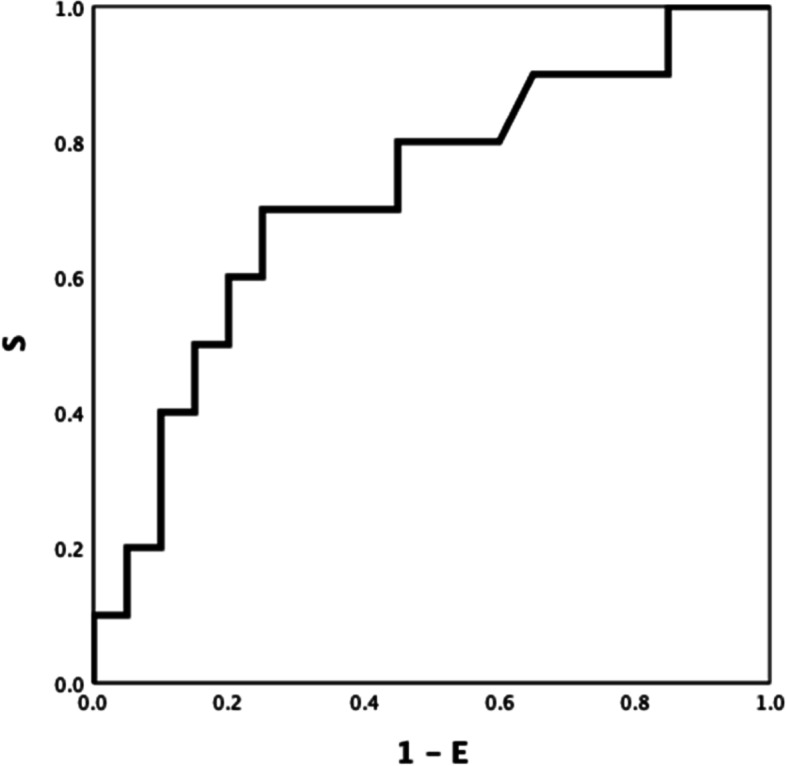
AD VS ACLF INTEM maxV-t 0.723

**Fig. 6:**
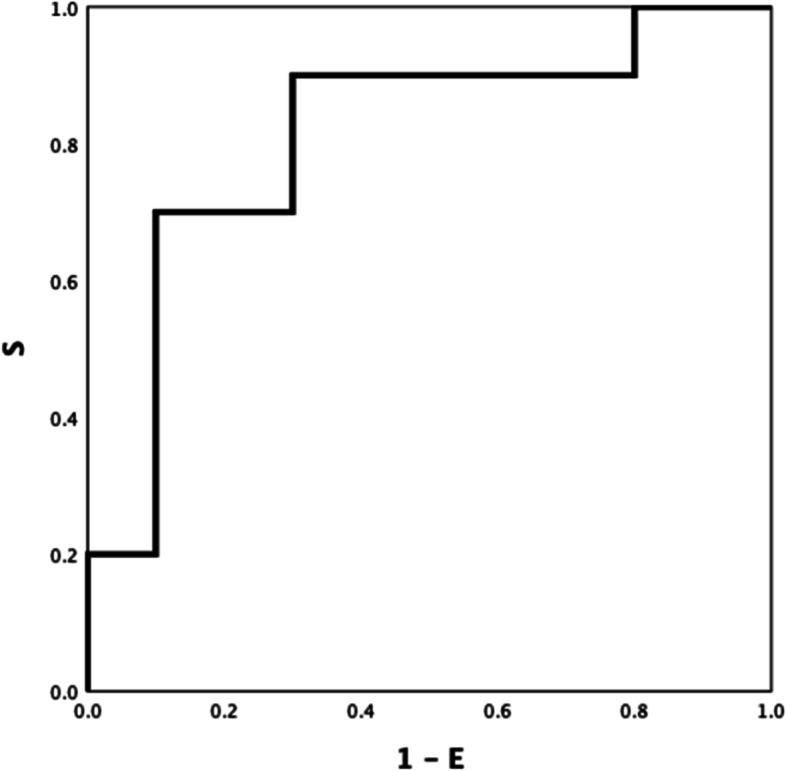
AD VS ACLF EXTEM LI60 + INTEM maxV-t 0.81

EXTEM LI60 and INTEM maxV-t differed between patients with AD and ACLF (ROC AUC of 0.743(Fig. 4), *P* = *0.033*; and 0.723, (Fig. 5) *P* = *0.050*; respectively). Discrimination improved moderately by combining both parameters, INTEM maxV-t + EXTEM LI60 (ROC AUC = 0.81) (Fig. 6) *P* < *0.001).*

### The addition of Protac® to blood samples did not improve the ability of standard, velocity, and fibrinolysis thromboelastometric tests performed without Protac® to identify the haemostasis pattern of patients with chronic liver disease from the haemostasis pattern of healthy controls

To investigate the potential benefit of Protac® in identifying the haemostasis pattern of patients with CLD, we repeated the ROC curve analysis including thromboelastometry tests performed with and without Protac®. None of the thromboelastometric parameters with Protac® discriminated better between patients and controls compared with the same assay and parameter performed without Protac® (data not shown). To further investigate the potential ability of Protac® to identify the haemostasis pattern of patients with CLD, we calculated the ROC curves of the ratio of all thromboelastometry parameters performed without and with Protac® (Table S2 Electronic Supplement). Overall, no significant improvement in the discrimination between patients with CLD and healthy controls was observed after the addition of Protac® to the standard thromboelastometry tests.

EXTEM LI60-ratio (inverse) and INTEM CFT-ratio (inverse), both with an optimal cut-off of 0.95, showed the best ROC AUC (0.824 and 0.817, respectively; both *P-values* < *0.001*). However, they did not increase the ability to define the haemostatic pattern of the same test performed without Protac® (Table S2 Electronic Supplement). The EXTEM LI60-ratio showed a sensitivity of 96% and the INTEM CFT-ratio a sensitivity of 90% in differentiating healthy controls from patients with CLD, but the specificity was very low (5%) in both cases (Table S2 Electronic Supplement, Table 4, Table S3 Electronic Supplement). We found no differences at baseline or after the addition of Protac® between patients who bled vs. those who did not.

**Table 4:** EXTEM and INTEM kinetic parameter (CT AND CFT) differences without and with Protac® challenge

**Differences in EXTEM AND INTEM kinetic parameters**	**Controls (*N* = 21)**	**AD + ACLF (*N* = 30)**	***P*-value (t-test)**
EXTEM ΔCT	16 (-8–21)	0 (-9–6)	0.01
EXTEM ΔCFT	7 (-1–22)	6 (-17–24)	0.33
INTEM ΔCT	-2 (-31–17)	15 (-18–28)	0.20
INTEM ΔCFT	12 (-0.5–24)	-12 (-34–2)	< 0.001

### Combination of thromboelastometric parameters for ROC curve analysis increases the ability to define the haemostatic pattern of patients with chronic liver disease

**Fig. 7:**
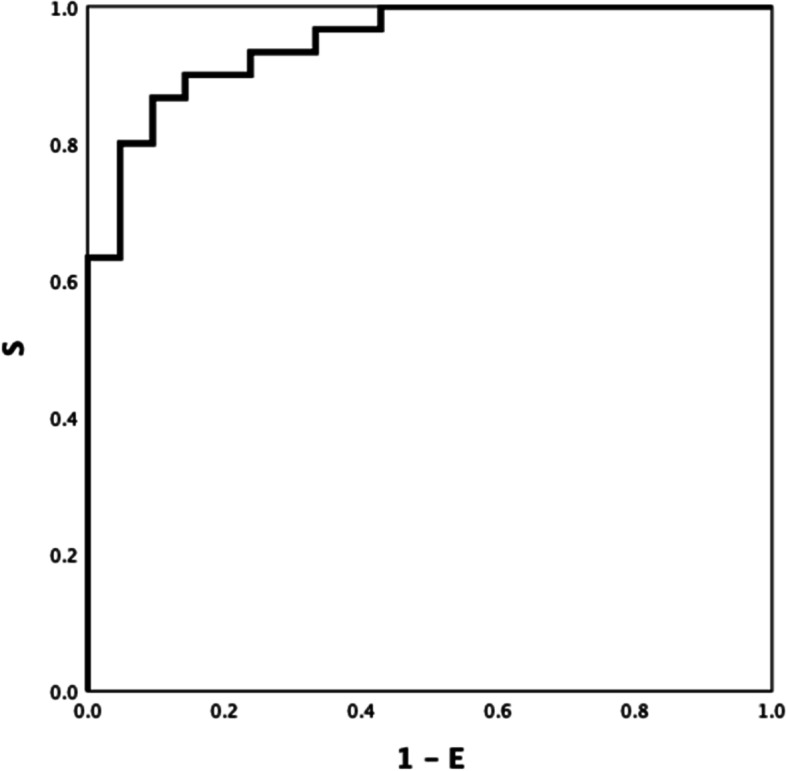
HEALTHY VS DISEASE INTEM CFT / EXTEM LI60-ratio 0.948

We combined data from standard ROTEM tests and ROTEM test modified by the addition of Protac® in order to improve the haemostasis pattern definition of patients with liver disease. The criteria for selecting combinations of thromboelastometric parameters was based on their clinical meaning. For example, we combined the velocity of clot formation with lysis, or firmness of clot with lysis, to include parameters that represent different physiologic steps of coagulation. The INTEM CFT/EXTEM LI60-ratio showed the highest ROC AUC 0.948 (*P* < *0.001*) (Fig. 7). Similarly, the INTEM CFT/EXTEM A5 x EXTEM LI60-ratio and INTEM CFT + EXTEM ML ratio both had a ROC AUC of 0.947 (both *P-values* < *0.001*). However, they only marginally increased the ability to identify the haemostasis pattern of CLD patients observed in EXTEM TT20 + INTEM CFT (AUC of 0.944; *P* < *0.001*) (Table 5). The best combination to define the haemostasis pattern of patients with AD from patients with ACLF was the combination of INTEM maxV-t + EXTEM LI60, which showed a ROC AUC of 0.81 (*P* < *0.001*) (Table 5).

**Table 5:** ROC curve analyses for combined ROTEM parameters to identify the coagulation pattern of patients with chronic liver disease and healthy controls

**ROTEM parameter**	**ROC AUC**	**95% CI**	***P*-VALUE**	**OPTIMUM** **CUT-OFF**	SENS	SPEC	PPV	NPV
**Healthy Controls (CON) vs. Liver Disease Patients (AD + ACLF)**								
EXTEM TT20 + INTEM CFT	0.944	0.884–1.000	< 0.001	0.254	96%	72%	83%	94%
EXTEM TT20 + EXTEM LI60	0.913	0.827–0.998	< 0.001	0.319	90	66	79	82
INTEM CFT + EXTEM LI60	0.927	0.852–1.00	< 0.001	0.329	90	89	87	85
EXTEM TT20 + INTEM CFT + EXTEM LI60	0.943	0.876–1.00	< 0.001	0.678	83	100	100	81
EXTEM TT20 – EXTEM A5	0.902	0.812–0.991	< 0.001	106	86	81	87	81
EXTEM TT20 / EXTEM A5	0.896	0.825–0.987	< 0.001	4.11	80	100	100	78
EXTEM TT20 / (EXTEM A5 x EXTEM ML)	0.881	0.775–0.987	< 0.001	0.780	80	85	87	78
INTEM CFT – INTEM A5	0.929	0.853–1.000	< 0.001	45.5	90	85	90	86
INTEM CFT / INTEM A5	0.929	0.855–1.000	< 0.001	2.571	80	95	96	77
INTEM CFT – 10 × INTEM TPI	0.926	0.850–1.000	< 0.001	67.6	90	85	87	85
INTEM CFT / INTEM TPI	0.925	0.849–1.000	< 0.001	50.1	90	81	87	85
EXTEM TT20 + EXTEM ML-ratio	0.929	0.852–1.000	< 0.001	0.571	80	90	91	79
EXTEM TT20 x EXTEM ML-ratio	0.778	0.639–0.918	0.001	53.21	80	58	94	67
INTEM CFT – INTEM ΔCFT	0.895	0.802–0.988	< 0.001	87.5	86	85	90	82
INTEM CFT / EXTEM LI60-ratio	0.948	0.893–1.000	< 0.001	89	90	85	90	82
INTEM CFT / EXTEM A5 x EXTEM LI60-ratio	0.947	0.885–1.000	< 0.001	2	90	85	87	89
INTEM CFT + EXTEM ML-ratio	0.947	0.887–1.000	< 0.001	0.587	80	95	95	80
INTEM CFT x EXTEM ML-ratio	0.919	0.841–0.998	< 0.001	97.39	80	90	100	63
INTEM CFT-ratio + EXTEM LI60-ratio	0.879	0.779–0.979	< 0.001	0.711	80	95	96	77
INTEM CFT-ratio x EXTEM LI60-ratio	0.170	0.045–0.295	< 0.001	0.662	80	0.04	56	17
1 / (INTEM CFT-ratio + EXTEM LI60-ratio)	0.121	0.021–0.221	< 0.001	1.211	43	0.04	39	6
1 / (INTEM CFT-ratio x EXTEM LI60-ratio)	0.838	0.720–0.956	< 0.001	0.973	80	85	59	41
**AD vs. ACLF**								
INTEM maxV-t – EXTEM ML	0.745	0.555–0.935	0.031	219	80	65	53	87
INTEM maxV-t / EXTEM ML	0.744	0.508–0.981	0.060	49	85	58	43	92
INTEM maxV-t + EXTEM LI60	0.81	0.636–0.984	0.006	0.324	80	70	57	87
INTEM maxV-t x EXTEM LI60	0.750	0.564–0.936	0.028	21,481	80	65	53	87
INTEM maxV-t – INTEM ΔCFT	0.713	0.531–0.894	0.062	191	90	55	37	83

## Discussion

This is the first study to assess the effect of adding Protac® to blood samples from healthy individuals and patients with CLD on thromboelastometric parameters. Significant impairment, mainly in kinetic parameters, was observed after adding Protac® to samples from healthy subjects, whereas this effect was negligible in patients with cirrhosis. We also show that specific combinations of standard, velocity, and fibrinolysis ROTEM parameters better define the haemostasis profile of patients with CLD than thromboelastometric assays performed with Protac®.

The anticoagulant pathway is underexplored in global tests currently used in clinical practice (ROTEM®, TEG®), which is their main limitation. *In-vivo*, thrombomodulin, located on the cell membrane of endothelial cells, activates protein C (the main anticoagulant driver). This important biological step should be reflected by assays including thrombomodulin to properly assess the overall haemostatic performance. Protein C activation can be replicated *in-vitro* by the snake venom Protac®. In our study, protein C was activated with Protac®, leading to hypocoagulability as reflected by prolonged kinetic and velocity ROTEM parameters in blood samples from healthy individuals treated with Protac® compared with those not treated. However, this effect was not seen in samples from patients with CLD. This confirms reports on thrombin generation tests performed with and without thrombomodulin, another way to activate protein C *in-vitro *[24]. As protein C levels are lower in patients with CLD compared with healthy individuals, the addition of thrombomodulin obviously has much less impact in patients with CLD. However, when thrombomodulin is added to thrombin generation assays, no differences in thrombin generation between patients and controls have been reported [23]. However, samples from patients with CLD remained hypocoagulable compared with those from healthy controls when we added Protac® to the thromboelastometric assay. Differences in the inherent nature of the tests (e.g., plasma vs. whole blood), and differences in protein C activation (thrombomodulin vs. Protac®) may be responsible for these differences [21, 34]. Likewise, thromboelastometry assesses fibrin formation, not thrombin generation, and deficiencies in fibrin polymerization (reported in patients with CLD) could result in an output other than that expected.

Activation of the anticoagulant pathway is particularly interesting in patients with CLD given the deficiency in both pro and anticoagulant factors seen in these patients: This was the rationale to include Protac® in the global test. Surprisingly, none of the thromboelastometric parameters performed with Protac® provided a better definition of the haemostasis profile of patients with CLD than the same parameter performed without Protac®. Again, issues related to the *in-vitro* activation of the anticoagulant pathway in the absence of endothelial cells may account for this. Furthermore, CFT and A5 in the INTEM test had already showed an excellent diagnostic performance (ROC AUC, 0.921 and 0.906 respectively; both *P-values* < *0.001*) in identifying haemostasis patterns in patients with cirrhosis (Table S1 Electronic Supplement) which is not easy to improve. Accordingly, TPI, a ROTEM index calculated using MCF and CFT, was the parameter that best defined the haemostasis profile of patients with CLD, revealing that these patients show slower clot formation (CFT) and, to a lesser extent, reduced clot firmness amplitudes (A). These changes are not proportional to the severity of CLD, pointing to a different haemostasis rebalance in AD than in ACLF patients. TPI could be assessed as a new predictor for bleeding or thrombotic complications, as well as to guide therapy in actively bleeding patients, in future clinical studies.

The combination of EXTEM TT20 + INTEM CFT (AUC 0.944; *P* < *0.001*) slightly increased the diagnostic performance of the TPI. EXTEM TT20 + INTEM CFT values over 0.254 identified patients with liver disease with a sensitivity of 96% and a specificity of 72% compared with healthy individuals. The addition of Protac® to the standard thromboelastometric parameters (e.g., EXTEM LI60-ratio with and without Protac®), as used in the ROTEM index INTEM CFT / EXTEM LI60-ratio, marginally increased the discriminative performance (ROC AUC 0.948; *P* < *0.001*) of the standard parameters but does not justify the inclusion of Protac® in thromboelastometric reagents.

Protac® decreased fibrinolysis when added to samples from healthy subjects (Table 3), and EXTEM LI60 values over 96% identified patients with ACLF with a sensitivity of 80% and specificity of 50%, vs. patients with AD. (Table S1 Electronic Supplement). This may be due to a decrease in platelet-mediated clot retraction, rather than an actual decrease in fibrinolytic activity, as Hartmann reported in 2020 [35], since FIBTEM ML does not change. On the other hand, hypofibrinolysis/fibrinolysis shutdown is suggested in patients with ACLF compared with patients with AD. It has been shown that higher values of EXTEM LI60, increased the risk of thrombosis and were associated to disease severity in critically ill COVID-19 patients [36, 37]. Protac® can activate platelet factor 4 which regulates whether the thrombin-thrombomodulin complex results in protein C or thrombin-activatable fibrinolysis inhibitor (TAFI) activation. High platelet factor 4 levels can result in decreased TAFI activation and hyperfibrinolysis [38]. Furthermore, the higher TPI values observed in samples from ACLF patients points to microthromboses as a potential mechanism of organ failure, as recently suggested [39]. However, it cannot excluded that the differences observed in EXTEM ML or LI60 after Protac® may be due to changes in platelet-mediated clot retraction rather than changes in fibrinolytic activity, since FIBTEM ML remained unchanged [35].

Besides the novelty, the main strength of this study is that samples from the same individual, drawn at the same time, were run in parallel with ROTEM assays with and without Protac®. The *in-vitro* setting always precludes definitive clinical conclusions and this is a limitation of the study. Due to the small sample size, the fibrinolysis parameter approach to discriminate patients with AD from those with ACLF is more hypothesis generating and has to be confirmed in a bigger cohort. Anyway, standard, velocity, and fibrinolysis ROTEM parameters, particularly if used in combination, already provide a useful definition of the haemostasis profile of patients with CLD.

Clinically, the most useful contribution is that TPI, a thromboelastometry parameter that summarizes the velocity of clot formation and clot firmness, could be used to assess the haemostatic potential of patients with cirrhosis, because it has been shown to be very different from that of healthy subjects. Conceptually, the anticoagulant pathway cannot be activated by thomboelastometry in patients with CLD.

In conclusion, Protac® as a protein C activator, was added to blood samples from healthy individuals and patients with CLD. A specific combination of standard, velocity, and fibrinolysis thromboelastometric parameters accurately defines the haemostasis profile of patients with CLD consisting of slow clot formation and reduced clot firmness. The addition of Protac® to the thromboelastometry test did not result in a better definition of the profile. A multicentre observational trial based on the data from this pilot study regarding complications is planned.

The findings suggest that haemostasis rebalance could change across the severity of liver disease, showing once again, that one size does not fit all, and that specific subgroups need to be defined to provide more precise and personalized treatment.

## Supplementary Information

**Additional file 1.**

## Data Availability

The datasets used and/or analysed during the current study are available from the corresponding author on reasonable request.
