# Supplementary material for: Haemostasis patterns in patients with acute-on-chronic liver failure and acute decompensation of cirrhosis including thromboelastometric tests with and without the addition of Protac: a pilot study
Source: Thromb J. 2022 Dec 13;20:75. doi: 10.1186/s12959-022-00438-3 (PMC9744590; doi:10.1186/s12959-022-00438-3)
Supplement: Supplementary file 1 — Additional file 1. [file 12959_2022_438_MOESM1_ESM.docx]

**Electronic Supplement**

**HAEMOSTASIS PATTERNS IN PATIENTS WITH ACUTE-ON-CHRONIC LIVER FAILURE AND ACUTE DECOMPENSATION OF CIRRHOSIS INCLUDING THROMBOELASTOMETRIC TESTS WITH AND WITHOUT THE ADDITION OF PROTAC: A PILOT STUDY**

Andrea Calvo, MD^1^, Miguel Angel Torrente, MD^2^, Klaus Görlinger, MD^3^, Javier Fernandez,MD, PhD^4^, Enric Reverter, MD, PhD^4^, Julia Vidal, MD^5^, Dolors Tassies MD, PhD^7^, Jordi Colmenero, MD, PhD^4^ Annabel Blasi, MD, PhD^6*^, Juan Carlos Reverter, MD, PhD^7*^.

*1.Anaesthesiology and Critical Care Department, Hospital Clínic, Institute d'Investigacions Biomédica Agust  Pi i Sunyer (IDIBAPS), University of Barcelona, Barcelona, Spain.*

*2. Haematology Department, Hospital Clínic, and University of Barcelona.*

*3. Department of Anaesthesiology and Intensive Care Medicine, University Hospital Essen, University Duisburg-Essen, Essen, Germany, and Medical Department, Tem Innovations GmbH, Munich, Germany.*

*4.Institut d’Investigacions Biomèdiques August Pi-Sunyer (IDIBAPS) y Ciber de Enfermedades Hepáticas y Digestivas (CIBEREHD), Liver Unit, Institut de Malalties Digestives i Metabòliques, Hospital Clínic, and University of Barcelona.*

*5. Anaesthesiology Department, Hospital Clínic, Barcelona, Spain.*

*6. Anaesthesiology Department, Hospital Clínic, and University of Barcelona, Spain, Institut d’Investigacions Biomèdiques August Pi-Sunyer (IDIBAPS) y Ciber de Enfermedades Hepáticas y Digestivas (CIBEREHD).*

*7. Haematology Department, Hospital Clínic, and University of Barcelona.*

* Senior *authorship shared.*

Corresponding author: Annabel Blasi. *Anaesthesiology Department, Hospital Clínic, and University of Barcelona, Spain, Institut d’Investigacions Biomèdiques August Pi-Sunyer (IDIBAPS) y Ciber de Enfermedades Hepáticas y Digestivas (CIBEREHD).* Postal code 08036. Phone: +34 932 27 5558; E-mail:   ablasi@clinic.cat

**CONTENTS**

1. **Tables**

**Table S1: ROC CURVE ANALYZES FOR STANDARD THROMBOELASTOMETRIC TESTS TO IDENTIFY THE COAGULATION PATTERN OF PATIENTS WITH CHRONIC LIVER DISEASE AND HEALTHY CONTROLS**

| **ROTEM PARAMETER** | **ROC AUC** | **95% CI** | **P-VALUE** | **OPTIMUM**  **CUT-OFF** | **SENS** | **SPEC** | **PPV** | **NPV** |
| --- | --- | --- | --- | --- | --- | --- | --- | --- |
| **HEALTHY CONTROLS vs. PATIENT WITH CHRONIC LIVER DISEASE (AD+ACLF)** | | | | | | | | |
| EXTEM CT | 0.788 | 0.658-0.915 | 0.001 | 56 | 76% | 60% | 77% | 62% |
| EXTEM CFT | 0.863 | 0.759-0.968 | <0.001 | 96 | 83% | 81% | 83% | 81% |
| EXTEM TT20 | 0.901 | 0.811-0.991 | <0.001 | 149 | 90% | 76% | 90% | 76% |
| EXTEM A5 | 0.100 | 0.012-0.188 | <0.001 | 43 | 20 | 23 | 27 | 17 |
| 1 / EXTEM A5 | 0.900 | 0.812-0.988 | <0.001 | 0.025 | 80 | 100 | 100 | 78 |
| EXTEM TPI | 0.110 | 0.014-0.205 | <0.001 | 1.17 | 33 | 5 | 33 | 4.8 |
| 1 / EXTEM TPI | 0.890 | 0.795-0.986 | <0.001 | 0.683 | 80 | 95 | 96 | 77 |
| EXTEM maxV | 0.171 | 0.057-0.285 | <0.001 | 12 | 36 | 19 | 39 | 17 |
| EXTEM LI60 | 0.741 | 0.606-0.877 | 0.004 | 92 | 80 | 47 | 69 | 62 |
| EXTEM ML (at 60 min after CT) | 0.259 | 0.123-0.394 | 0.004 | 3.5 | 40 | 15 | 40 | 14 |
| ΔLI60 (FIB-EX) | 0.263 | 0.120-0.405 | 0.004 | -0.9 | 93 | 5 | 56 | 20 |
| INTEM CFT | 0.921 | 0.845-0.997 | <0.001 | 82 | 90 | 77 | 84 | 84 |
| INTEM TT20 | 0.882 | 0.776-0.987 | <0.001 | 304 | 80 | 96 | 96 | 77 |
| INTEM A5 | 0.094 | 0.003-0.185 | <0.001 | 39 | 20 | 10 | 24 | 8 |
| 1 / INTEM A5 | 0.906 | 0.815-0.997 | <0.001 | 0.026 | 80 | 100 | 96 | 77 |
| INTEM TPI | 0.072 | 0.000-0.147 | <0.001 | 1.3 | 20 | 15 | 24 | 8 |
| 1 / INTEM TPI | 0.928 | 0.853-1.000 | <0.001 | 0.842 | 80 | 100 | 100 | 78 |
| INTEM maxV | 0.138 | 0.033-0.243 | <0.001 | 10 | 53 | 5 | 44 | 7 |
| **AD (0) vs. ACLF (1)** | | | | | | | | |
| EXTEM CFT | 0.640 | 0.414-0.866 | 0.218 | 66 | 80 | 65 | 80 | 65 |
| EXTEM L60 | 0.743 | 0.553-0.932 | 0.033 | 96 | 80 | 50 | 80 | 50 |
| EXTEM ML | 0.258 | 0.068-0.447 | 0.033 | 0.5 | 70 | 5 | 70 | 5 |
| 1 / EXTEM ML | 0.677 | 0.450-0.904 | 0.174 | 0.225 | 85 | 52 | 40 | 90 |
| ΔLI60 (FIB-EX) | 0.295 | 0.096-0.494 | 0.071 | -0.5 | 80 | 10 | 80 | 10 |
| FIBTEM AUC | 0.370 | 0.158-0.582 | 0.253 | 866 | 90 | 20 | 90 | 20 |
| INTEM maxV-t | 0.723 | 0.523-0.922 | 0.050 | 238 | 70 | 75 | 70 | 75 |

**TABLE S2: ROC CURVE ANALYSES FOR RATIOS WITHOUT AND WITH PROTAC® CHALLENGE TO IDENTIFY COAGULATION PATTERN OF PATIENTS WITH CHRONIC LIVER DISEASE AND HEALTHY CONTROLS**

| **ROTEM PARAMETER** | **ROC AUC** | **95% CI** | **P-VALUE** | **OPTIMUM**  **CUT-OFF** | **SENS** | **SPEC** | **PPV** | **NPV** |
| --- | --- | --- | --- | --- | --- | --- | --- | --- |
| **HEALTHY CONTROLS vs. CHRONIC LIVER DISEASE PATIENTS (AD+ACLF)** | | | | | | | | |
| EXTEM CT-ratio | 0.325 | 0.162-0.487 | 0.034 | 0.86 | 76 | 29 | 61 | 46 |
| EXTEM CFT-ratio | 0.420 | 0.262-0.578 | 0.334 | 0.92 | 73 | 15 | 55 | 27 |
| EXTEM TT20-ratio | 0.294 | 0.150-0.439 | 0.013 | 0.92 | 63 | 24 | 54 | 31 |
| EXTEM A5-ratio | 0.613 | 0.460-0.767 | 0.171 | 0.91 | 70 | 43 | 61 | 47 |
| EXTEM maxV-t-ratio | 0.416 | 0.258-0.573 | 0.130 | 0.88 | 66 | 24 | 56 | 33 |
| EXTEM LI60-ratio | 0.176 | 0.052-0.300 | <0.001 | 0.99 | 96 | 5 | 59 | 50 |
| 1 / EXTEM LI60-ratio | 0.824 | 0.700-0.948 | <0.001 | 0.95 | 86 | 53 | 71 | 71 |
| EXTEM ML-ratio | 0.703 | 0.550-0.857 | 0.018 | 0.36 | 73 | 58 | 59 | 50 |
| FIBTEM CT-ratio | 0.452 | 0.291-0.612 | 0.559 | 1.01 | 70 | 24 | 57 | 36 |
| FIBTEM ML-ratio | 0.611 | 0.298-0.925 | 0.505 | 0.23 | 55 | 80 | 83 | 50 |
| FIBTEM AUC-ratio | 0.454 | 0.291-0.616 | 0.579 | 0.73 | 80 | 24 | 60 | 45 |
| INTEM CT-ratio | 0.624 | 0.465-0.783 | 0.135 | 0.86 | 86 | 44 | 63 | 60 |
| INTEM CFT-ratio | 0.183 | 0.058-0.307 | <0.001 | 0.56 | 90 | 5 | 57 | 25 |
| 1 / INTEM CFT-ratio | 0.817 | 0.693-0.942 | <0.001 | 0.95 | 80 | 71 | 80 | 71 |
| INTEM TPI-ratio | 0.659 | 0.504-0.813 | 0.056 | 0.78 | 86 | 43 | 67 | 67 |
| INTEM AUC-ratio | 0.297 | 0.150-0.443 | 0.014 | 0.94 | 73 | 10 | 54 | 20 |
| **AD vs. ACLF** | | | | | | | | |
| EXTEM LI60-ratio | 0.445 | 0.234-0.656 | 0.628 | 1.00 | 50 | 45 | 33 | 66 |
| 1 / EXTEM LI60-ratio | 0.500 | 0.275-0.725 | 1.00 | 0.00 | 100 | 0 | 35 | 65 |
| EXTEM ML-ratio | 0.350 | 0.102-0.597 | 0.248 | 0.43 | 57 | 32 | 24 | 67 |
| INTEM CFT-ratio | 0.512 | 0.307-0.718 | 0.912 | 0.88 | 70 | 50 | 39 | 75 |
| INTEM TPI-ratio | 0.555 | 0.350-0.670 | 0.628 | 1.00 | 90 | 50 | 47 | 90 |
|  | | | | | | | | |

**TABLE S3: ROC CURVE ANALYSES OF EXTEM AND INTEM KINETIC PARAMETER (CT AND CFT) DIFFERENCES WITHOUT AND WITH PROTAC® CHALLENGE**

| **DIFFERENCES IN EXTEM AND INTEM KINETIC PARAMETER** | **ROC AUC** | **95% CI** | **P-VALUE** | **OPTIMUM**  **CUT-OFF** | **SENS** | **SPEC** | **PPV** | **NPV** |
| --- | --- | --- | --- | --- | --- | --- | --- | --- |
| **HEALTHY CONTROLS vs. PATIENTS WITH CHRONIC LIVER DISEASE (AD+ACLD)** | | | | | | | | |
| EXTEM ΔCT | 0.337 | 0.175-0.498 | 0.049 | -8.5 | 76 | 24 | 46 | 30 |
| EXTEM ΔCFT | 0.437 | 0.278-0.596 | 0.450 | -18 | 76 | 5 | 53 | 12 |
| INTEM ΔCT | 0.609 | 0.448-0.769 | 0.190 | -24.5 | 86 | 34 | 65 | 63 |
| INTEM ΔCFT | 0.199 | 0.069-0.329 | <0.001 | -14.5 | 56 | 10 | 70 | 71 |
| -INTEM Δ CFT | 0.81 | 0.671-0.931 | <0.001 | -4.5 | 90 | 74 | 81 | 75 |
| **HEALTHY CONTROLS vs. AD** | | | | | | | | |
| EXTEM ΔCT | 0.362 | 0.185-0.539 | 0.130 | -6.5 | 80 | 29 | 52 | 60 |
| EXTEM ΔCFT | 0.452 | 0.265-0.640 | 0.602 | -1 | 60 | 24 | 43 | 38 |
| INTEM ΔCT | 0.676 | 0.509-0.844 | 0.054 | -1 | 75 | 53 | 60 | 69 |
| INTEM ΔCFT | 0.238 | 0.074-0.403 | 0.004 | -49 | 70 | 5 | 41 | 14 |
| -INTEM ΔCFT | 0.762 | 0.597-0.926 | 0.004 | -4.5 | 90 | 70 | 73 | 79 |
| **HEALTHY CONTROLS vs. ACLF** | | | | | | | | |
| EXTEM ΔCT | 0.286 | 0.088-0.483 | 0.057 | -7 | 60 | 29 | 29 | 60 |
| EXTEM ΔCFT | 0.407 | 0.149-0.665 | 0.410 | -18 | 80 | 5 | 29 | 33 |
| INTEM ΔCT | 0.474 | 0.246-0.701 | 0.816 | -24 | 70 | 44 | 33 | 70 |
| INTEM ΔCFT | 0.121 | 0.000-0.255 | 0.001 | -28 | 90 | 5 | 31 | 50 |
| -INTEM ΔCFT | 0.879 | 0.745-1.000 | 0.001 | -6 | 90 | 80 | 56 | 93 |
| **AD vs. ACLF** | | | | | | | | |
| EXTEM ΔCT | 0.363 | 0.140-0.585 | 0.226 | -17 | 80 | 10 | 31 | 50 |
| EXTEM ΔCFT | 0.488 | 0.253-0.722 | 0.912 | -18 | 80 | 25 | 35 | 71 |
| INTEM ΔCT | 0.350 | 0.122-0.578 | 0.187 | -21.5 | 70 | 10 | 28 | 40 |
| INTEM ΔCFT | 0.520 | 0.316-0.724 | 0.860 | -22.5 | 80 | 40 | 40 | 80 |
| -INTEM ΔCFT | 0.480 | 0.276-0.684 | 0.860 | -20.5 | 60 | 30 | 36 | 72 |
